# Concussion Reduction in Division I and II Athletes: Effects of Simple Cervical Spine Exercise Regimen

**DOI:** 10.7759/cureus.66058

**Published:** 2024-08-03

**Authors:** Joel Klein, Ian Koch, Blake E Delgadillo, Jason Chickness, Jacob Blank, Ashton Amos, Kevin Tay, Emily A Kelly, Kassidy Webber, Brett Benzinger, Jeffrey Haft, Drew Miller

**Affiliations:** 1 Physiatry, Rochester Regional Health, Rochester, USA; 2 Orthopedic Surgery, Lake Erie College of Osteopathic Medicine, Erie, USA; 3 Orthopedic Surgery, Lake Erie College of Osteopathic Medicine, Bradenton, USA; 4 General Surgery, Lake Erie College of Osteopathic Medicine, Erie, USA; 5 Athletic Training, Mercyhurst University, Erie, USA

**Keywords:** head impact, concussion prevention, cervical range of motion, orthopedic sports medicine, neck muscle strength, brain concussion, collegiate athlete, sport-related injury, sport-related concussion, pericervical muscle

## Abstract

Introduction: Primary preventative medicine lacks a consensus on effective concussion prevention strategies for collegiate athletes. Cervical strength has been identified as a potential factor in concussion risk reduction. This study evaluates the impact of a commercially available, portable cervical muscle stretching and strengthening device, NeckX®, on cervical strength, range of motion (ROM), and concussion incidence in collegiate athletes participating in high-concussion-risk sports.

Methods: A single-arm prospective cohort study was conducted with 162 collegiate athletes from various sports. Participants underwent a 12-week neck exercise protocol using the NeckX® device. Clinical data, including neck strength and ROM, were collected at weeks 0, 6, and 12. Concussion incidence was self-reported by participants and cross-referenced with records from the athletic department. Data were analyzed for significant neck strength and ROM changes throughout the 12-week study. A two-way analysis of variance multiple comparisons with the Tukey-Kramer significant difference test was utilized, using the Holm-Sidak method, with an alpha of 0.05.

Results: All athletic teams experienced a significant increase in cervical strength during the 12-week intervention (α = 0.05, p < 0.05). Increases in cervical flexion and extension force were most consistent between teams. Cervical ROM increased significantly in male and female soccer players (α = 0.05, p < 0.05). The overall incidence of head and neck injuries, including concussions, was reduced to 6.60% during the study period, the lowest recorded value in the university’s athletic department history.

Conclusion: The use of the NeckX® device for 12 weeks was effective in enhancing pericervical muscle strength and ROM while reducing concussion incidence in collegiate athletes participating in high-concussion-risk sports. Interestingly, the positive outcomes were consistent for both males and females, indicating the universal advantages of neck training among collegiate athletes. These findings support existing research on the benefits of cervical strengthening exercises for reducing concussions in collegiate athletes and highlight the convenience and affordability of using this device.

## Introduction

Sports-related concussions (SRCs) are underreported, inadequately managed, and often not identified in collegiate athletes [1]. Primary preventative medicine does not provide a clear picture of how to prevent concussion in these individuals [2,3]. Straightforward solutions, such as improvements in protective equipment, have been predominantly ineffective in reducing concussions in competition [2,4]. The National Collegiate Athletic Association’s (NCAA) concussion safety protocol largely focuses on concussion education, protective equipment, and management once an athlete is concussed but provides no evidence-based protocols for concussion prevention [5]. Young athletes with multiple concussions suffer acutely from shorter and less prosperous careers in professional sports [6] and, in the long term, with increased development of chronic traumatic encephalopathy and associated neurodegenerative disorders [7-9].

Yengo-Kahn et al. found that a single concussion costs approximately $800 in healthcare system spending for high school athletes [10]. Athletes who developed postconcussion syndrome have higher initial symptom burdens and incur a four-time greater average cost compared with those without the syndrome [10]. Importantly, it is worth noting that as many as 10% of concussed athletes experience symptoms (e.g., headache, sleep disturbances, and balance problems) that persist beyond two weeks [11]. The management teams for these athletes are often multidisciplinary, providing guidance and monitoring athletes for weeks to months after injury [12,13].

Neck strength has been identified as a potential factor in reducing concussion risk as well as neck pain [14-16]. Despite this evidence, there is a lack of comprehensive studies examining the impact of targeted neck strengthening programs on concussion incidence and pericervical muscle function in male and female collegiate athletes. Many previous studies have concentrated on elite male athletes, often with limited diversity in sports. In this study, we aimed to assess the effectiveness of a commercially available, low-cost, portable, and user-friendly pericervical exercise device. We wanted to determine its potential to enhance cervical muscle strength and range of motion (ROM) and reduce SRCs. Importantly, our investigation involved collegiate athletes participating in various sports, encompassing both male and female athletes.

## Materials and methods

Participants

One hundred seventy-five (175) collegiate athletes aged ≥18 years (range: 18-25 years) participating in high-concussion-risk sports within NCAA Divisions I and II were recruited for the study between August 2021 and January 2022. The research included individuals from Mercyhurst University, a North American institution, participating in various sports such as men’s hockey, women’s lacrosse, men’s and women’s soccer, and men’s wrestling. Before data collection, a physician screened the subjects for eligibility, using exclusion criteria that consisted of age <18 years at initiation, a lifetime history of cervical pathology (e.g., spondylolisthesis, fracture, and degenerative disc disease), previous cervical spine surgery, recent cervical trauma within the last six months, or moderate-to-severe pain with cervical range of motion (ROM) evaluation. Ethical approval was obtained from the Mercyhurst University Institutional Review Board, and all subjects provided written informed consent before participation. Participants consented by reading and signing our participation consent document, which outlined the study’s associated risks, the research objectives, and the procedures that would be carried out.

A total of 162 subjects (89 male, 73 female) successfully completed the study. Two subjects voluntarily withdrew from the study for unspecified personal reasons, and 10 subjects were excluded from the study due to failure to complete at least 50% of the workout protocol. Notably, no subjects withdrew from the study due to neck injury associated with the device or complications related to the protocol.

Study design

**Figure 1:**
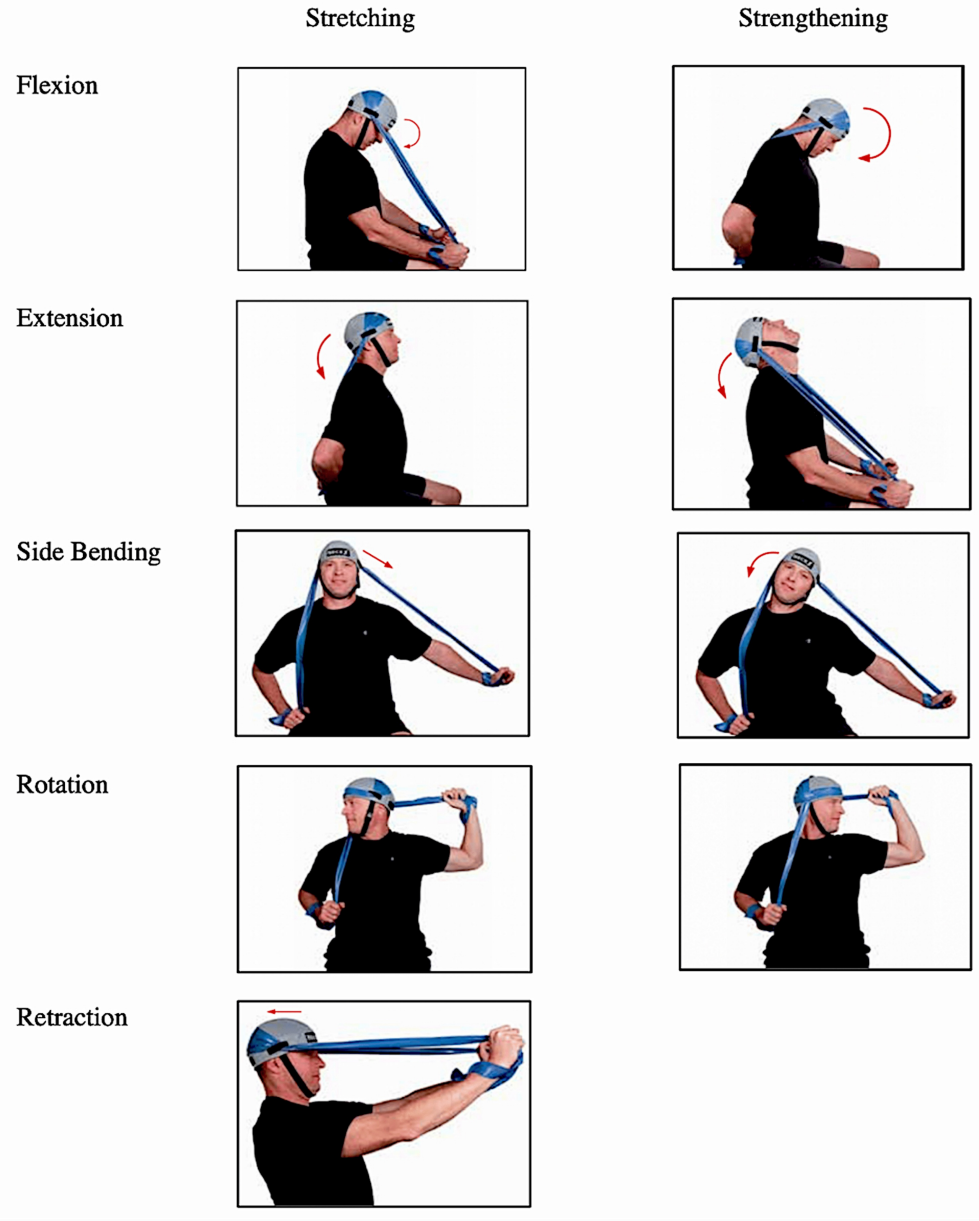
Stretching and strengthening movements using NeckX® device This image was used with permission from NeckX® [17]. The individual shown was not a study participant

This study employed a single-arm prospective cohort design to evaluate subjects for changes in pericervical muscle strength, cervical ROM, and concussion incidence over a 12-week period. The participants followed a three-session-per-week routine, engaging in a novel cervical stretching and strengthening protocol utilizing the NeckX® device (NeckX® LLC, Aspen, CO) (Table 1, Figure 1). Each session was designed to last approximately 10 minutes. Pericervical muscle strength and ROM were measured at weeks 0, 6, and 12. Successful subject completion of the study was defined as subjects completing at least 50% of the workout protocol during the study period, as self-reported by the participants. All subject data were stored securely on an encrypted database, accessible only by the research team in a desensitized form. Study subjects and data collectors were blinded to previous measurement data during data collection and post-study analysis.

**Table 1:** Exercise protocol reps: repetitions; yellow: lowest resistance; green: medium resistance; blue: highest resistance

Band color/exercise	Week 1	Week 2	Week 3	Weeks 4-6
Therapy band color	Yellow	Green	Blue	Blue
Stretching (1 rep = 10 seconds of passive stretch)				
Flexion	2 reps	2 reps	2 reps	2 reps
Extension	2 reps	2 reps	2 reps	2 reps
Side bending	2 reps right, 2 reps left	2 reps right, 2 reps left	2 reps right, 2 reps left	2 reps right, 2 reps left
Neck rotation	2 reps right, 2 reps left	2 reps right, 2 reps left	2 reps right, 2 reps left	2 reps right, 2 reps left
Strengthening				
Flexion	10 reps × 2 sets	10 reps × 2 sets	10 reps × 2 sets	10 reps × 2 sets
Extension	10 reps × 2 sets	10 reps × 2 sets	10 reps × 2 sets	10 reps × 2 sets
Retraction	10 reps × 2 sets	10 reps × 2 sets	10 reps × 2 sets	10 reps × 2 sets
Side bending	10 reps × 2 sets right, 10 reps × 2 sets left	10 reps × 2 sets right, 10 reps × 2 sets left	10 reps × 2 sets right, 10 reps × 2 sets left	10 reps × 2 sets right, 10 reps × 2 sets left
Neck rotation	10 reps × 2 sets right, 10 reps × 2 sets left	10 reps × 2 sets right, 10 reps × 2 sets left	10 reps × 2 sets right, 10 reps × 2 sets left	10 reps × 2 sets right, 10 reps × 2 sets left

Clinical evaluation

The study’s primary outcome was concussion incidence, with secondary outcomes including pericervical muscle strength and ROM. Concussion incidence was self-reported by each subject and cross-referenced with total concussion incidence reported by each team’s athletic trainer, obtained from electronic records maintained by the Athletic Health Department at Mercyhurst University. Records were reviewed for concussions sustained in competition by each participating athletic team over the past eight years. Concussions were confirmed using the ImPACT software (ImPACT Applications, Inc., Coralville, IA) from 2014 to 2019 and the SWAY medical software (Sway Medical, Inc., Tulsa, OK) from 2020 to 2022.

Pericervical strength was assessed using the Activ5® dynamometer (Activbody, Inc., San Diego, CA) in six planes of motion: cervical flexion, extension, side bending (right and left), and rotation (right and left). Subjects performed a single five-second and gradually increasing maximal output repetition in each plane of motion against a counterforce provided by a researcher. Pericervical strength was measured at weeks 0, 6, and 12, with strict adherence to a seated upright position during data collection to minimize confounding exertional forces and recruitment of muscle groups aside from those in the cervical spine.

Cervical ROM was measured using a Halo digital goniometer (Halo Medical Devices, LLC, Sydney, Australia). Subjects sat in a neutral spine position and performed three repetitions of cervical flexion, extension, side bending (right and left), and rotation (right and left). The Halo device was attached to the subjects’ heads for accurate measurements.

Study protocol

The 162 subjects who completed the study participated in three data collection events at weeks 0, 6, and 12 and adhered to at least 50% completion of the 12-week workout protocol using the NeckX® device (Table 1). Over the 12-week period, participants performed three 10-minute weekly workout sessions at their preferred location (e.g., home, gym, and school). Our research team provided participants with comprehensive instructions on how to perform the exercises before they received the device. The workout included five minutes of cervical stretching and five minutes of cervical strengthening activities. The protocol was progressive, with increased repetitions and stronger therapy bands implemented as subjects progressed through the 12-week study.

Statistical analysis

Clinical pericervical muscle strength and ROM values were statistically analyzed and reported as means and standard deviations. Statistical comparisons were conducted between the data recorded at weeks 0, 6, and 12 for each respective category using a two-way analysis of variance (ANOVA) and Tukey’s multiple comparisons test. An alpha of 0.05 was utilized without assuming a consistent standard deviation. The analysis was performed using the GraphPad Software, LLC version Prism 9.3.1.

## Results

One hundred seventy-five (175) student-athletes from Mercyhurst University in North America were initially recruited for this study during the 2021-2022 athletic season. However, 13 subjects were excluded from data analysis due to preexisting limitations, inadequate compliance (<50%) with the exercise protocol, or elective withdrawal from the study for reasons unrelated to the research. Ultimately, the final statistical analysis included 162 athletes, and their demographic characteristics are presented in Table 2.

**Table 2:** Subjects’ demographic characteristics

Gender	Number of participants	Mean age (range 18-25 years)	Mean height (inches)	Mean weight (pounds)
Male	89	20.6	71.1	181.3
Female	73	19.5	63.7	133.8
Total	162	20.1	67.6	156.5

Pericervical muscle strength

**Figure 2:**
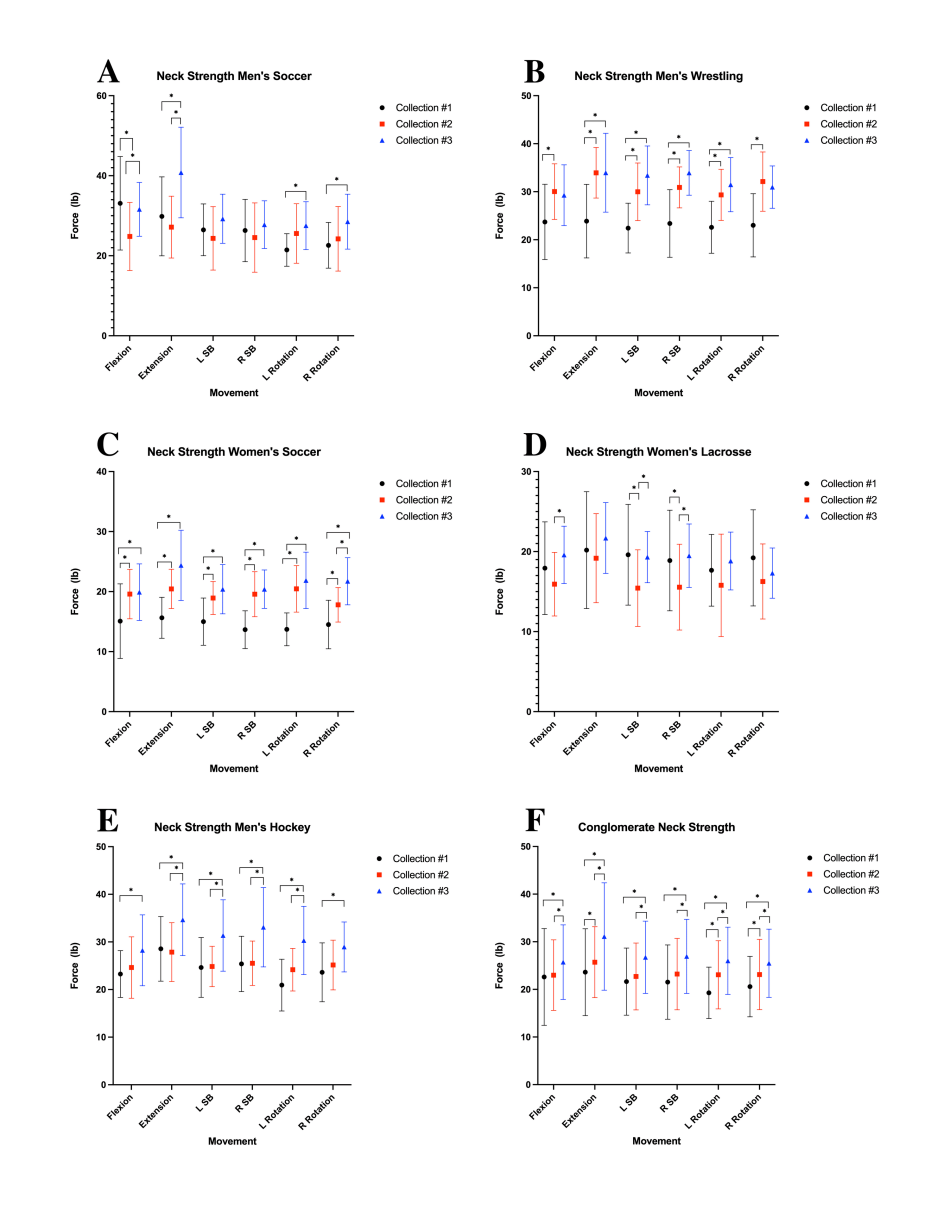
Pericervical strength measurements for (A) men’s soccer, (B) men’s wrestling, (C) women’s soccer, (D) women’s lacrosse, (E) men’s hockey, and (F) conglomerate data from all participants lb: pound; L SB: left side bending; R SB: right side bending; L rotation: left rotation; R rotation: right rotation; *: statistically significant increases calculated using multiple comparison analysis of variance (α = 0.05, p < 0.05); collection #1: week 0 measurement collection; collection #2: week 6 measurement collection; collection #3: week 12 measurement collection

Across all athletic teams, a significant increase in conglomerate pericervical muscle strength was observed in at least one six-week period during the study. The most common period of increased strength was between weeks 6 and 12, occurring in four out of five teams (Figure 2). Significant increases in pericervical muscle strength were observed across various sports teams. For men’s soccer, there were significant increases in mean cervical flexion strength between collections 1-2 and 2-3 (p < 0.0001, 0.0038), mean extension strength between collections 1-3 and 2-3 (p < 0.0001, 0.0001), mean left rotation strength during collection 1-3 (p = 0.0100), and mean right rotation strength during collection 1-3 (p = 0.0126) (Figure 2). Men’s wrestling exhibited an increase in mean cervical flexion strength during collection 1-2 (p = 0.0172), mean extension strength between collections 1-2 and 1-3 (p < 0.0001, 0.0103), mean left side bending strength between collections 1-2 and 1-3 (p = 0.0032, 0.0046), mean right side bending strength between collections 1-2 and 1-3 (p = 0.0034, 0.0068), mean left rotation strength between collections 1-2 and 2-3 (p = 0.0099, 0.0278), and mean right rotation strength during collection 1-2 (p = 0.0003). Women’s soccer athletes demonstrated increases in mean cervical flexion strength during collections 1-2 and 1-3 (p < 0.0001, 0.0005), mean extension strength during collections 1-2, 1-3, and 2-3 (p < 0.0001, 0.0001, 0.0061), mean left side bending strength between collections 1-2 and 1-3 (p = 0.0008, 0.0001), mean right side bending strength during collections 1-2 and 1-3 (p < 0.0001, 0.0001), mean left rotation strength during collections 1-2 and 1-3 (p < 0.0001, 0.0001), and mean right rotation strength between collections 1-2, 1-3, and 2-3 (p = 0.0067, 0.0001, 0.0059). Women’s lacrosse saw significant increases in mean cervical flexion strength during collection 1-2 (p = 0.0366), mean left side bending strength during collections 1-2 and 2-3 (p = 0.0040, 0.0248), and mean right side bending strength during collections 1-2 and 2-3 (p = 0.0283, 0.0222). Male hockey players experienced increases in mean cervical flexion strength during collection 1-3 (p = 0.0291), mean extension strength during collections 1-3 and 2-3 (p = 0.0051, 0.0022), mean left side bending strength between collections 1-3 and 2-3 (p = 0.0018, 0.0037), mean right side bending strength between collections 1-3 and 2-3 (p = 0.0003, 0.0005), mean left rotation strength between collections 1-3 and 2-3 (p < 0.0001, 0.0066), and mean right rotation strength during collection 1-3 (p = 0.0176). The mean recorded conglomerate force measurements for the various movements across all the different teams are illustrated in Figure 2. This single graph illustrates the changes in cervical strength over three time points: week 0, week 6, and week 12 (collections 1, 2, and 3).

Cervical range of motion

**Figure 3:**
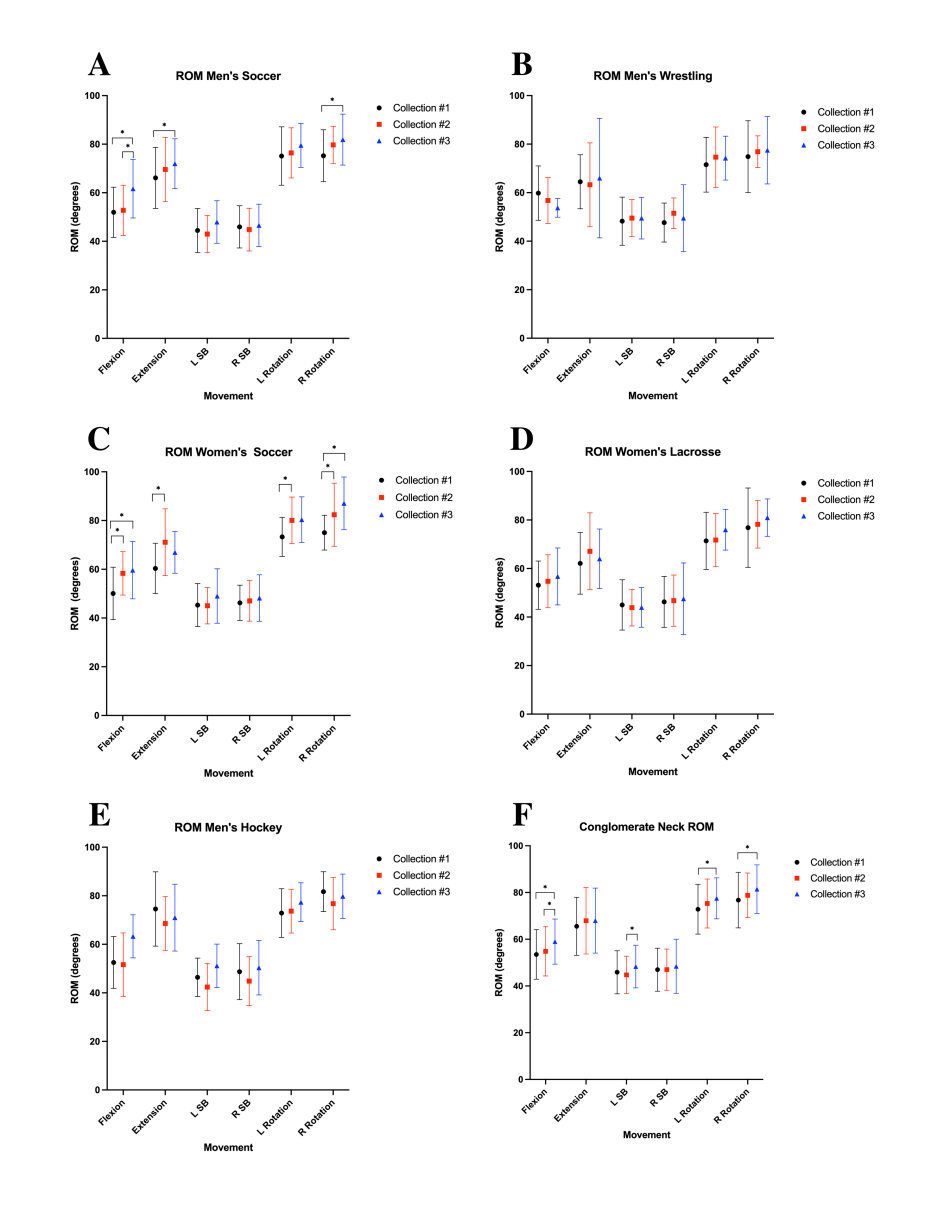
Pericervical ROM for (A) men’s soccer, (B) men’s wrestling, (C) women’s soccer, (D) women’s lacrosse, (E) men’s hockey, and (F) conglomerate data from all participants ROM: range of motion; L SB: left side bending; R SB: right side bending; L rotation: left rotation; R rotation: right rotation; *: statistically significant increases calculated using multiple comparison analysis of variance (α = 0.05, p < 0.05); collection #1: week 0 measurement collection; collection #2: week 6 measurement collection; collection #3: week 12 measurement collection

The cervical range of motion (ROM) was variably impacted by the study’s cervical training protocol. The most notable increase in ROM among the various sports teams occurred primarily during weeks 6-12 (Figure 3). For men’s soccer, there were significant increases in mean cervical flexion ROM during collections 1-3 and 2-3 (p = 0.0003, 0.0041), mean extension ROM during collection 1-3 (p = 0.0472), and mean right rotation ROM during collection 1-3 (p = 0.0194) (Figure 3). Women’s soccer demonstrated increases in mean cervical flexion ROM during collections 1-2 and 1-3 (p = 0.0060, 0.0072), mean extension ROM during collection 1-2 (p = 0.0002), mean left rotation ROM during collection 1-2 (p = 0.0299), and mean right rotation ROM during collections 1-2 and 1-3 (p = 0.0166, 0.0005). However, no significant changes in mean cervical ROM were observed in men’s hockey, men’s wrestling, and women’s lacrosse (Figure 3). The mean recorded conglomerate ROM measurements for all the various movements across all the different teams are illustrated in Figure 3. This single graph illustrates the changes in cervical ROM over three time points: week 0, week 6, and week 12 (collections 1, 2, and 3).

Flexion/extension ratio

The flexion/extension (F/E) strength ratios were calculated using the cervical flexion and extension forces collected at 0, 6, and 12 weeks. The team F/E ratios ranged from 0.77 to 1.10, while the F/E ratio combining all participants ranged from 0.82 to 0.99. Throughout the study, F/E ratios decreased from 0.91 at week 0 data collection to 0.86 at week 12 data collection for female athletes and from 0.98 at week 0 data collection to 0.80 at week 12 data collection for male athletes (Figure 3).

Male versus female athletes

Female athletes exhibited mean cervical strength output ranging from 60.1% to 73.0% of male athletes, with otherwise unremarkable variability around respective means. ROM means and ranges were nearly identical between the sexes, and concussion incidence did not show significant differences between male and female athletes.

Concussion

The incidence of concussions among participating teams during the 2021-2022 season was 6.60% per athletic event. This represented the lowest recorded value in the past eight athletic seasons (2014-2022) at Mercyhurst University (Table 3).

**Table 3:** Concussion incidence per athletic season from 2014 to 2022

Totals/concussion incidence	2014-2015	2015-2016	2016-2017	2017-2018	2018-2019	2019-2020	2020-2021	2021-2022
Total concussions	11	24	13	13	8	8	6	7
Total games/matches	117	107	111	106	107	93	52	105
Concussion incidence	9.4	22.43	11.71	12.26	7.47	8.6	11.54	6.67

## Discussion

There is a need to validate concussion-mitigating techniques to supplement the use of personal protective equipment (PPE) in high-risk athletes. Annually, there are approximately 1.6-3.8 million concussions, also known as mild traumatic brain injuries, associated with sports and recreational activities, with athletes and military personnel being most susceptible [18]. The biomechanics of concussive injuries are intimately associated with head acceleration, velocity, and displacement. Even small reductions in head velocity are associated with reduced concussions [2,4,19]. PPE, such as padded helmets, often falls short in the prevention of concussions due to factors such as head velocity [19]. This study presents a primary prevention strategy to decrease the incidence of SRCs in male and female athletes across multiple sports. The main conclusions from this investigation are as follows: (1) a simple protocol for strengthening the cervical spine is effective in quantitatively increasing the mean neck strength across various planes of motion in collegiate athletes and (2) increasing the cervical strength of collegiate athletes appears to be correlated with a decreased incidence of head and neck injuries, including SRCs, sustained during competitive play. After evaluating historical concussion data, we observed that the incidence (6.67%) of head and neck injuries during the period of the study was the lowest recorded in the university’s history (Table 3).

The findings of this study support the implementation of a neck-strengthening protocol as a primary preventative measure against concussion. However, it is worth noting that the women’s lacrosse and men’s soccer teams paradoxically experienced nonsignificant reductions in overall strength between study weeks 0 and 6. This outcome may be misleading as it was likely attributable to trunk mispositioning during the initial strength data collection, an issue corrected before gathering the week 6 data. F/E ratios prove useful in determining the legitimacy of cervical strength data due to the consistency of human anatomy [20,21]. The men’s soccer team had an F/E ratio of 1.109 during the first data collection, deviating significantly from the acceptable values in the literature (0.5-0.9), suggesting inadequate trunk stabilization [20]. During the first data collection, the cervical force measurements were collected while the athletes of these two teams sat on the bleachers without any back support. This was corrected by incorporating straight-backed chairs for the remainder of the study. As a result, the F/E ratios from that point forward ranged from 0.77 to 0.99, considerably closer to the acceptable ranges described in the literature of 0.50-0.90 [20]. Similar studies utilizing trunk-stabilizing devices reported F/E ratios ranging from 0.65-0.73 in elite rugby players [21]. Additionally, previous studies have demonstrated sex-specific reliability in measuring maximal isometric cervical muscle strength using handheld dynamometry [22].

Research has indicated that every one-pound increase in neck strength corresponds to a 5% decrease in concussion risk [23]. A systematic review by Elliot et al. revealed that greater neck strength, combined with participation in injury prevention exercises, including those targeting the neck, lowers the likelihood of athletes sustaining head or neck injuries, including SRCs [24]. This aligns with the present study’s results.

Additionally, Collins et al. demonstrated that decreased mean neck circumference, decreased mean head-to-neck circumference ratio, and decreased neck strength all correlated with an increased risk of sustaining a concussion [23]. Although the subject population of the Collins study was high school athletes rather than collegiate athletes, the findings are consistent with the data presented here, highlighting the potential of increased neck strength as a primary preventative measure against SRCs [23]. In contrast to the previously discussed studies, a systematic review of literature by Daly et al., encompassing elite male rugby and American football players, revealed insufficient evidence to establish a correlation between an increase in neck strength and a reduction in concussion incidence [25]. However, the data presented in this current investigation would likely improve the power and generalizability of the study by Daly et al. [25].

The increased cervical extension strength found near-universally across all subjects mirrors the findings of Anderson et al. [14]. This study also found a decrease in concussion incidence, affirming earlier findings by Farley et al. that highlighted the notable link between cervical extension strength and a decrease in concussions among professional rugby players [26]. Notably, female athletes tend to have higher concussion rates than male athletes [27]. Nonetheless, research on collegiate, professional, and elite athletes often overlooks female athletes or includes a limited variety of sports. This study is more inclusive of sports and sex by studying women’s and men’s soccer, men’s ice hockey, women’s lacrosse, and men’s wrestling. The results underscore that sex did not play a role in the ability to increase neck strength from this protocol. Therefore, for groups at higher risk of SRCs, such as female athletes, performing these neck exercises could help lower the risk of sustaining an SRC.

Limitations

Several limitations to the present study warrant consideration in interpreting the findings. Some of these limitations occurred by design, including the self-reported compliance used to exclude participants. Self-reported compliance may help streamline participant selection based on their commitment to the study protocol, but it also has the potential to introduce a source of bias. The accuracy is likely influenced by memory recall and personal perceptions. Therefore, participants reporting their engagement in the study could deviate from actual participation, potentially affecting the reliability of the data. By relying on self-reported compliance, a level of subjectivity may be introduced.

Furthermore, the study was performed on a cohort of young, healthy, and spine-surgery-naive patients who reported little to no cervical pain before enrollment. Thus, the generalizability of this study’s conclusions may be limited to a younger athletic population without preexisting cervical pathology.

Another limitation of this investigation is the absence of a control group. Although this study did compare the outcomes to previous athletic seasons in which neck stretching and strengthening were not utilized, such a methodology does constitute a control group. Future research should include a control group to further elucidate the findings of this study. Furthermore, the lack of blinding and a placebo group may induce bias. These are additional areas that future research may wish to consider in order to enhance control.

An unforeseen limitation arose during the initial data collection (week 0) due to a lack of trunk stabilization, as described previously. The cervical flexion measurements, specifically in women’s lacrosse and men’s soccer during week 0, were likely falsely increased due to trunk mispositioning during the initial data collection. The initial F/E ratio of 1.109 recorded for the men’s soccer team during the first data is much higher than the accepted range of 0.5-0.9, indicating potential inadequate trunk stabilization. This discrepancy likely emanated from the absence of straight-backed chairs during the initial data collection with the first two teams (men’s soccer and women’s lacrosse). Straight-backed chairs were used for the rest of the athletic teams during week 0 and throughout the rest of the study. Therefore, the lack of trunk stabilization affected only these two teams’ cervical strength outcomes during week 0.

## Conclusions

This study delved into the impact of a 12-week neck-training regimen involving a low-cost, portable, and user-friendly cervical exercise device. This product proved efficacious in reducing concussion incidence, enhancing cervical ROM, and improving pericervical strength in collegiate athletes. Significant improvements in pericervical strength were noted throughout the study among the athletic teams, with the most notable enhancements observed during the 6-12 week timeframe. Although some teams did not have significant outcomes, especially with regard to improvements in cervical ROM, the collective outcomes of the study indicated a positive trend in improving both pericervical muscle strength and ROM. This study found few distinctions between male and female athletes’ responses to this intervention, which may imply its universal applicability in athletic populations. Moreover, the study aligns with previous literature, emphasizing the importance of cervical strengthening as a preventative measure against SRCs. The incidence of concussions among participating teams during the 2021-2022 season was 6.60% per athletic event, which was the lowest value in university history. Future studies evaluating the efficacy of similar neck-strengthening protocols would greatly benefit from including comprehensive control groups to validate these results.
